# Risk, Adultification, Messaging, and Protection Scale (RAMPS): The development of a measure for Black girls

**DOI:** 10.1111/jora.70123

**Published:** 2026-01-15

**Authors:** Natasha Crooks, Nyssa Snow‐Hill, Abigail Bushnell, Kimberly Sanker‐Panchal, Gina Sissoko

**Affiliations:** ^1^ University of Illinois Chicago Chicago Illinois USA; ^2^ DePaul University Chicago Illinois USA; ^3^ Yale University New Haven Connecticut USA

**Keywords:** adultification, Black girls, measurement, messaging, protection, sexual behavior

## Abstract

Adultification, protection, and stereotyping of Black girls and their associated sexual and reproductive health risks are understudied concepts that are challenging to measure. This study developed and validated the Risk, Adultification, Messaging, and Protection Scale (RAMPS), designed to assess the relationship between sexual development, adultification, messaging, and protection, which are constructs of the *Becoming a Sexual Black Woman* framework. Preliminary items were derived from qualitative interviews with Black girls aged 9–18 years old. Adolescent responses (*N* = 575) to the RAMPS were subjected to several analyses to evaluate the measure's psychometric properties. Exploratory and confirmatory factor analyses (CFA) guided by the *Becoming a Sexual Black Woman* theory were used to test item fit; correlations between the refined measures and theoretically relevant measures were examined for validity; and measurement invariance of the RAMPS was evaluated across three age groups. CFA demonstrated a strong fit for a 3‐factor model. The interpretation of the measure was fully invariant across age groups. Findings indicated that the RAMPS represents a valid framework for measuring factors impacting Black girls' sexual development. The final 12‐item measure demonstrated respectable to very good internal consistency—adultification (α = .81, very good), protection (α = .76, respectable), messaging (α = .78, respectable), and total protection (α = .80, very good). This paper presents psychometric information about the RAMPS and the full set of items. The next steps will be to validate the measure within a larger sample and to explore its correlation with sexually transmitted infections and HIV risk.

## INTRODUCTION

Black girls experience inequities in HIV and sexually transmitted infections (STIs) in the United States (U.S.). In 2022, Black adolescents aged 13–24 accounted for 50% of new HIV infections in the U.S. (Centers for Disease Control and Prevention, 2023). Adolescents with a history of STIs are at increased risk of HIV. Among adolescent girls aged 11 to 19 years old, HIV risk is nearly 5 times as high for those with three or more STIs (e.g., chlamydia) (Newbern et al., 2013). Among adolescent females aged 15–19 years, Black girls are 5 times more likely to get chlamydia compared to white girls (Centers for Disease Control and Prevention, 2020). The average age of first sexual intercourse for Black girls is 15 years, compared to 16 years for white girls (Biello et al., 2013). Additionally, Black girls are disparagingly impacted by sexual objectification and violence due to adultification (Crooks et al., 2023; Crooks, King, & Tluczek, 2022), as one in every four Black girls will be sexually abused before the age of 18, indicating a greater need to protect them (Brinkman et al., 2024; Klot, 2013). All these factors inherently increase Black girls' risk for HIV and STIs. Thus, it is imperative to understand what contributes to or protects Black girls against early sexual engagement, sexual violence, and HIV/STI risk among Black girls (Centers for Disease Control and Prevention, 2020; Crooks, King, & Tluczek, 2022; Crooks, Sosina, et al., 2022).

Most research examining HIV/STI risk and sexual behavior among Black girls has used an epidemiological perspective where race is viewed as a risk or protective factor, and racial groups are considered homogeneous or stratified by broad social factors, including socioeconomic status (Andu et al., 2018; Brown, 2008; Caldwell et al., 2004; Townsend, 2008). Racial identity is a critical component that has been associated with Black girls' sexual development (e.g., sexual attitudes and risk behaviors) in the literature (Garcia‐Reid et al., 2020; Opara et al., 2022; Townsend, 2008). Racial identity is often correlated with self‐esteem and pride, which inherently relates to sexual behaviors and decision‐making among Black girls. The intersection of racial and sexual identities involves navigating both racism and sexism, which can impact community, mental well‐being, and perceptions of self. However, HIV/STI programming and interventions tend to use cognitive behavioral theories developed without consideration of more nuanced individual differences from the perspectives of Black girls (Crooks & Muehrer, 2019). The CDC suggests that sociocultural factors are likely to account for the profound disparities of HIV/STIs in racial and ethnic minority females (Centers for Disease Control and Prevention, 2020). Sociocultural factors may include, but are not limited to, factors in psychosocial, physical, or cultural environments (Crooks, King, & Tluczek, 2022). Therefore, to alleviate sexual health disparities, it is necessary to understand the sociocultural factors (i.e., impoverished and violent living conditions, relationships, families, peers, and societal messaging) of the target population to design culturally grounded measures and interventions.

The *Becoming a Sexual Black Woman (BSBW)* is a theoretical framework describing how sociocultural factors influence Black girls' sexual development (Crooks et al., 2019). The BSBW framework highlights two major sociocultural factors, protection and stereotype messaging, that influence the trajectory of Black female sexual development as they traverse through the Girl, Grown, and Woman phases (Crooks et al., 2019). Protection is described as a means to prevent early sexualization and adultification of Black girls' sexuality and a key factor influencing Black female sexual development (Crooks et al., 2019, 2020). Levels of protection vary at different phases of development. Stereotype messaging consists of sexual messages that Black girls and women receive historically, culturally, and societally from the media. The BSBW describes three phases of sexual development (Girl, Grown, and Woman) occurring along two pathways (i.e., fast versus cautious) regarding sexual activity (Crooks, King, & Tluczek, 2022). The Girl phase is marked by early sexual development beginning around 9 years old and is described as a particularly vulnerable period (Crooks et al., 2019). Participants described differing risks for girls who are “looking grown,” “acting grown,” or “being grown” (Crooks et al., 2023). Girls who are looking or acting grown were identified as being more susceptible to early sexual engagement and HIV/STIs due to their development of menses, sexual characteristics (i.e., breasts and curvaceous figures), behaving older than their age, or adultification (Crooks et al., 2023; Epstein et al., 2017). Adultification refers to the contextual, social, and developmental processes that prematurely, and often inappropriately, expose children to adult‐like roles and responsibilities and assume adult‐like knowledge (Epstein et al., 2017; Thompson, 2020). Establishing protection and safe sexual behaviors during this period is important for Black girls' health and development. Yet, there is a critical gap in culturally tailored measures/instruments and interventions for Black girls.

More specifically, no effective measures exist to screen for early sexual engagement and HIV/STI risk in Black girls. HIV/STI screening measures used in routine health care settings are outdated, lack clinical relevance, and do not account for sociocultural factors and context (i.e., living conditions, influence of peers, sexual health information, and sexual trauma). Conceptually grounded measures that go beyond individual‐level risk can more effectively assess trends in sexual health and identify targets for early intervention (Crooks & Muehrer, 2019). Developing a measure grounded in a framework that aligns with the sexual development of Black girls, such as BSBW, can address this challenge. The purpose of this study was to develop and validate a measure of adultification, stereotype messaging, and protection that can be correlated with overall risk profiles of Black girls.

## METHOD

### Participants

There were 956 participants originally recruited to participate in this study. Participants were removed from the dataset if they opened the link but did not start the survey, resulting in 667 participants. Five participants were removed due to providing celebrity names (*n* = 662). Participants were removed if they completed the survey in <7 min, as this was determined to be the quickest time that someone could competently complete the survey (*n* = 580). One participant was removed because they did not answer any of the test items for the proposed measure (*n* = 579), and an additional four participants were removed because they were missing 75% of all the test items (*n* = 575). All participants identified as female and Black. Of the 575 participants, 83 were between the ages of 9 and 12, 178 were between the ages of 13 and 15, and 312 were between the ages of 16 and 18. The sample was split using a random number generator to be placed in one of two groups. The first split sample (*n* = 287) was used to conduct exploratory factor analysis (EFA). The second split sample (*n* = 288) was used to conduct confirmatory factor analysis (CFA).

### Procedures

A pool of 38 items was generated from qualitative interviews with 25 Black girls who participated in a study about Black girl development, aiming to accurately capture phases of sexual development (i.e., looking, acting, and being grown, protection, and stereotype messaging) (Crooks et al., 2024). We then conducted cognitive interviews with a different set of 20 Black girls to further refine and consult on the measurement items (Castillo‐Díaz & Padilla, 2013; Irwin et al., 2009). The resulting items reflected the theoretical model developed from these analyses (Crooks et al., 2019) and utilized direct wording frequently used by participants. An additional nine items were added based on feedback from girls, for a total of 43 items. These items were then evaluated in a REDCap survey with a larger sample of Black girls, which is the focus of this study.

Two recruitment methods were used: (1) community engagement, including snowball sampling through schools and community organizations, and (2) social media ads. Initially, we partnered with community partners (i.e., UI Health, Girls Inc. Chicago, Boys and Girls Club) in Chicago, who serve as stakeholders in Black girls' health and social care initiatives. We placed our study flyers in community partners' facilities, newsletters, and websites. Despite efforts, we only gained 53 participant responses from our community partners. After 8 months, we transitioned our recruitment efforts to social media ads, which involved recruitment on Facebook and Instagram, where participants' family members would be presented with an ad and could then introduce their girls to the study. We used social media to reach our target sample of 300 participants in <1 month. We also opened recruitment throughout the U.S. Those eligible for the study were encouraged to click the ad, and those with an eligible teen girl were prompted to share the ad with the teen. The images in the ads were selected to appeal to parents/caregivers and girls. The entire recruitment period lasted 10 months.

Eligibility criteria included self‐identifying as a Black girl, 9–18 years old, fluent in speaking and reading English, and living in the United States. Participating teen girls and their caregivers provided voluntary informed assent and consent electronically. Girls aged 9–17 provided an email address or phone number for their parents to be sent REDCap links to review study information and provide electronic parental permission, and girls then completed electronic assent. Girls aged 18 reviewed the study information, completed a brief screening questionnaire, provided electronic consent, and completed the survey. Girls and parents were assured of the confidentiality of the information provided. Participants were asked to complete a survey in English, which took approximately 15–20 min, and received a $15 Amazon e‐gift card for participating. All procedures were approved by the institutional review board at University of illinois Chicago.

### Measures

#### Multidimensional inventory of Black identity‐teen (MIBI‐t)

The MIBI‐t is a 21‐item measure comprised of seven subscales used to assess stable dimensions (Centrality, Regard, Ideology) of Black/African American racial identity in teens (Scottham et al., 2008). Only Centrality, Public Regard, and Private Regard were evaluated for this study. Participants respond to each item using a 5‐point scale ranging from “really disagree” to “really agree.” Item scores are averaged to create subscale composite scores. Using the Spearman‐Brown Formula, the authors found reliable internal consistencies of each subscale. The Centrality Scale (α = .78) measures how salient race is to an individual's identity. Higher scores indicate that race is a more central component of one's identity. The Regard scale includes the Private and Public subscales. The Private Regard subscale (α = .87) measures respondents' feelings toward other Blacks and their inclusion in that racial group. The Public Regard subscale (α = .79) measures how individuals think outgroup members view Blacks. Higher scales on both subscales indicate a more positive perspective toward Blacks. Internal consistencies in the current sample were as follows: Centrality (α = .72), Private Regard (α = .79), and Public Regard (α = .83).

#### Body appreciation scale‐2 (BAS‐2)

The BAS‐2 is a 10‐item measure assessing positive opinions toward one's body (Tylka & Wood‐Barcalow, 2015). Participants respond to whether a question is true about themselves using a 5‐point scale ranging from “never” to “always.” The authors found a Cronbach's coefficient of .94 for female respondents. Internal consistency in the current sample was 0.90.

#### Adolescent clinical sexual behavior inventory‐self‐report (ACSBI‐S)

The ACSBI‐S is a 45‐item assessment of sexual risk behaviors in adolescents (Friedrich et al., 2004). Participants respond on a 3‐point scale ranging from “not true” to “very true” about sexual behaviors in the past 12 months. Friedrich et al. (2004) reported a Cronbach's alpha of .86. Internal consistency in the present sample was 0.95.

#### NIH Toolbox Friendship Survey

The NIH Toolbox Friendship Survey is a 5‐item self‐report that measures perceptions of friendships in youth 8–17 years old (Salsman et al., 2013). Items are answered on a 5‐point scale from “never” to “always.” Higher scores indicate a higher perception of having friends to engage with. Internal consistency in the present sample was 0.80.

#### NIH Toolbox Emotional Support Survey

NIH Toolbox Emotional Support Survey is a 7‐item measure assessing perceived emotional support (Salsman et al., 2013). Items are answered on a 5‐point scale from “never” to “always”. High scores indicated higher emotional support. Internal consistency in the present sample was 0.89.

#### NIH Toolbox Maternal Relationship Survey

The NIH Toolbox Maternal Relationship Survey is a 3‐item measure that assesses youths' perception of closeness and meaningful time spent with their mother (National Institutes of Health, 2012a). Items are answered on a 5‐point scale from “never” to “always,” and one item is answered on a 4‐point scale ranging from “extremely close” to “not very close.” Internal consistency in the present sample was 0.77.

#### NIH Toolbox Paternal Relationship Survey

The NIH Toolbox Paternal Relationship Survey is a 3‐item measure that assesses youths' perception of closeness and meaningful time spent with their father (National Institutes of Health, 2012b). Items are answered on a 5‐point scale from “never” to “always,” and one item is answered on a 4‐point scale ranging from “extremely close” to “not very close.” Internal consistency in the present sample was 0.79.

#### Pubertal development scale (PDS)

The PDS‐female version is a 5‐item self‐report measure that assesses pubertal development (Carskadon & Acebo, 1993). Participants respond to each item using a 4‐point scale ranging from “not yet started” to “seems complete,” and one item asking, “Have you begun to menstruate?” is answered on a 2‐point scale: “yes” or “no.”

### Data analysis

#### Exploratory and confirmatory factor analysis

Analyses were conducted using Mplus 8.10. The total sample was randomly split such that half of the sample (*n* = 287) was used for exploratory factor analysis (EFA) while the other half of the sample (*n* = 288) was used for confirmatory factor analysis (CFA). Prior to conducting EFA, poor performing items were identified and removed if they had low item‐scale correlations (<0.4) or unexpected correlations among items (Monahan et al., 2009). EFA was conducted in a random half of the sample (*n* = 287) using robust maximum likelihood (MLR) estimation to handle any non‐normality in the test items. The rate of missingness for total responses was minimal, approximately 0.03%. Since the rate of missingness was minimal, full information maximum likelihood (FIML) was employed to estimate model parameters for all information and cases to be used in analyses. Geomin (oblique) rotation was specified to allow for correlations among the factors. Principal components analysis, interpretability of factors, number of items per factor, and theoretical fit to the model previously identified (i.e., Crooks et al., 2019) were used as criteria for determining the optimal factor structure (DeVellis & Thorpe, 2021; Furr, 2020). After determining the optimal factor structure, items with low factor loadings (<0.5; Hair et al., 2019) and items with cross loadings (>0.32; Comrey & Lee, 2013; Tabachnick & Fidell, 2001) were removed. Item performance was assessed, and redundant items were removed to produce a parsimonious final measure.

The final EFA factor structure was validated via CFA with a geomin rotation, MLR estimation, and FIML with the remaining half of the sample (*n* = 288). The following guidelines were used to assess model fit: (1) standardized root‐mean‐square residual (SRMR) <0.08 was acceptable and <0.05 was good; (2) root‐mean‐square error of approximation (RMSEA) <0.08 was acceptable and <0.05 was good; and (3) comparative fit indices (CFI) >0.90 were acceptable and >0.95 were considered good (Browne & Cudeck, 1992; Hu & Bentler, 1999).

#### Validation of Measure

Measurement invariance was evaluated across the three age groups (i.e., 9–12, 13–15, and 16–18) and utilized the full sample (*n* = 565), as has been used in previous measure development and validation studies (e.g., Waddell et al., 2022). The age ranges of 9–12, 13–15, and 16–18 were selected based on sociocultural factors and developmental phases of Girl, Grown, and Woman outlined by BSBW, which is the theoretical framework guiding this study. This framework provides distinctions between looking, acting, and being grown that are indicated by developmental landmarks such as the onset of menarche, physical maturation, and sexual engagement, which vary by age and are crucial to understanding Black girls' behaviors (Crooks et al., 2019, 2023). Measurement invariance was assessed in three steps. First, configural invariance was tested by comparing the pattern of loadings within each group. In configural invariance, no equality constraints are imposed across groups, and a good fit suggests that the basic factor structure is the same across groups. Second, metric invariance was tested by comparing the configural invariance model to a model where the factor loadings are constrained to be equal across groups to test if the relationship between the latent and observed variables is consistent. Third, scalar invariance was tested by constraining the intercepts (i.e., means of the observed indicators) to be equal across groups to evaluate if the items have the same meaning and are measuring the same construct at the same level across groups. Significant decrement in model fit was determined by change in three model fit indices: RMSEA, CFI, and SRMR. If changes between models are greater than 0.01 for CFI, 0.015 for RMSEA, and 0.03 for SRMR, measurement invariance across groups is not supported (Chen, 2007; Cheung & Rensvold, 2002). Measurement invariance analyses used listwise deletion for missing data on the grouping variable (*n* = 3), as Mplus does not allow FIML on missing data for grouping variables. To evaluate construct validity, correlations were conducted on SPSS with measures of constructs within the expected nomological network. For any missing data, imputed means were inserted.

## RESULTS

### Exploratory and confirmatory factor analysis

#### Exploratory factor analysis

**FIGURE 1:**
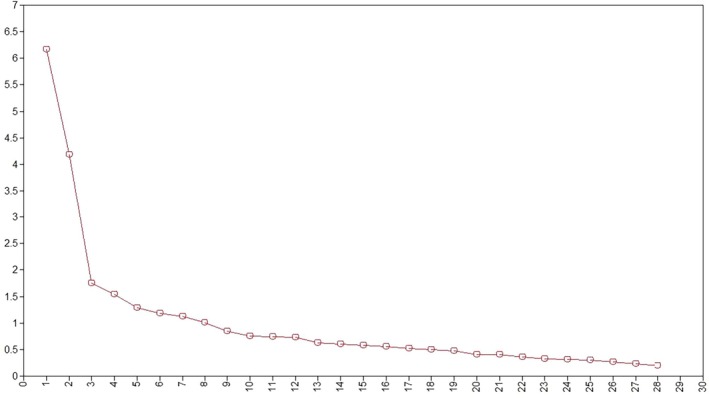
Scree plot of eigenvalues.

Participants (*n* = 287) provided responses to 38 items assessing risk for initial item selection. Ten items were removed due to low item‐scale correlations (<0.40). The remaining 28 items were analyzed using EFA in random sample 1 (*n* = 287). PCA identified 7 factors with eigenvalues greater than 1 (Kaiser, 1960). Upon consideration of the scree plot, there was not a clear “elbow” with which the progression of factors had a point where the information suddenly shifted from vertical to horizontal (Cattell, 1966). With a gentler curve, as is seen in Figure 1, factor interpretability is needed to determine the appropriate number of factors (DeVellis, 2017). Factor solutions with six or more factors had factors with ≤2 items with significant loadings and were not considered further. The one and two factor solutions did not fit the data well. Therefore, factor solutions with three, four, and five factors were considered.

Although a 4‐factor model had slightly better fit indices compared to a 3‐factor model, it introduced unnecessary complexity to the model by creating a factor that seemed to combine items that had similar language around cat calling, bullying, and harassment rather than creating a factor capturing a similar construct. These four items were removed from further analysis as it was determined that these items reflected knowledge about why cat calling, bullying, and harassment are risky as opposed to identifying aspects of whether a girl may be at risk or protected. The 5‐factor model showed only marginal improvement in fit indices (compared to the 4‐factor model) and may be overfitting the model as the fifth extracted factor appeared to be capturing random noise. Thus, the most parsimonious model with adequate fit was the 3‐factor model, which also aligns with the proposed model identified by Crooks et al. (2019). For further item refinement, ten items were iteratively removed for factor loadings below 0.5 (Hair et al., 2006), resulting in 14 remaining items: Factor 1 (adultification; 4 items); Factor 2 (protection; 6 items); and Factor 3 (messaging; 4 items).

#### Confirmatory factor analysis

A CFA was conducted with random sample 2 (*n* = 288) to examine factor loadings on a three‐factor model (i.e., adultification, protection, and messaging) with the preliminary 14‐item measure. Fit indices demonstrated that the 3‐factor model fit the data well (SRMR = 0.05 (good); RMSEA = 0.05 (good); CFI = 0.96 (good); *χ* ^2^ = 127.28, *p* < .05). All items maintained factor loadings above 0.5, except for one on the protection factor (0.49). To produce a parsimonious measure, the items on the protection factor were further analyzed. It was determined that two of the items (including the item with a slightly lower factor loading) read more like checklists on an inventory as opposed to being dimensions of the construct, and thus, were removed.

The final 12‐item measure performed well with a three‐factor model (i.e., adultification, protection, and messaging; SRMR = 0.05 (good); RMSEA = 0.05 (good); CFI = 0.96 (good); *χ* ^2^ = 100.56, *p* < .05). Although the chi‐square was still significant, chi‐square is not the best fit index for this sample as chi‐square has limitations and can be affected by the model. Higher scores on each of the subscales indicate greater experience of that construct. Although greater focus was paid to the individual subscales, a total score was derived to capture an overall measure of sexual risk protection, which included reverse scoring adultification. The final 12‐item measure demonstrated respectable to very good internal consistency – adultification (α = .81, very good), protection (α = .76, respectable), messaging (α = .78, respectable), and total protection (α = .80, very good). The 12‐item measure will, from here on, be referred to as the Risk, Adultification, Messaging, and Protection Scale (RAMPS). See Table 1 for individual item performance on the final 12‐item measure. See Figure 1 for a visual representation of the three‐factor solution with item loadings and factor variances.

**TABLE 1:** Item performance for the final 12‐items (*n* = 288).

Item	Construct represented	Factor loading	*R* ^2^	Mean	Standard deviation
1. I believe my body look grown (i.e., my body looks older than my age)	Adultification	0.74	.54	3.65	1.37
2. Based on my body shape, people tell me I look grown	Adultification	0.77	.60	3.78	1.10
3. Acting grown reflects my behaviors (i.e., I act older than my age)	Adultification	0.64	.41	3.64	0.93
4. Based on my behaviors, people tell me I act grown	Adultification	0.82	.67	3.95	0.94
5. I talk to my parents about my body, sexuality, and sexual behaviors	Protection	0.66	.44	3.92	1.14
6. My parent/guardian has taught me how to protect my body	Protection	0.63	.39	4.18	0.72
7. My parent/guardian responds well when I talk to them about my body	Protection	0.82	.68	4.0	0.86
8. In general, I feel like my body is protected	Protection	0.59	.34	4.04	0.66
9. Social media (i.e., Instagram, Facebook, TikTok) influences the way I behave positively (i.e., self‐esteem, confidence, social support)	Messaging	0.57	.33	3.82	0.82
10. Social media (i.e., Instagram, Facebook, TikTok) influences the way I feel about my body positively	Messaging	0.59	.35	3.95	0.76
11. Media (i.e., music, movies, TV) influences the way I behave positively	Messaging	0.81	.66	3.91	0.79
12. Media (i.e., music, movies, TV) influences the way I feel about my body positively	Messaging	0.70	.49	3.72	0.83

*Note*: The response scale was 1–5, with higher scores representing more of its respective factor.

**FIGURE 2:**
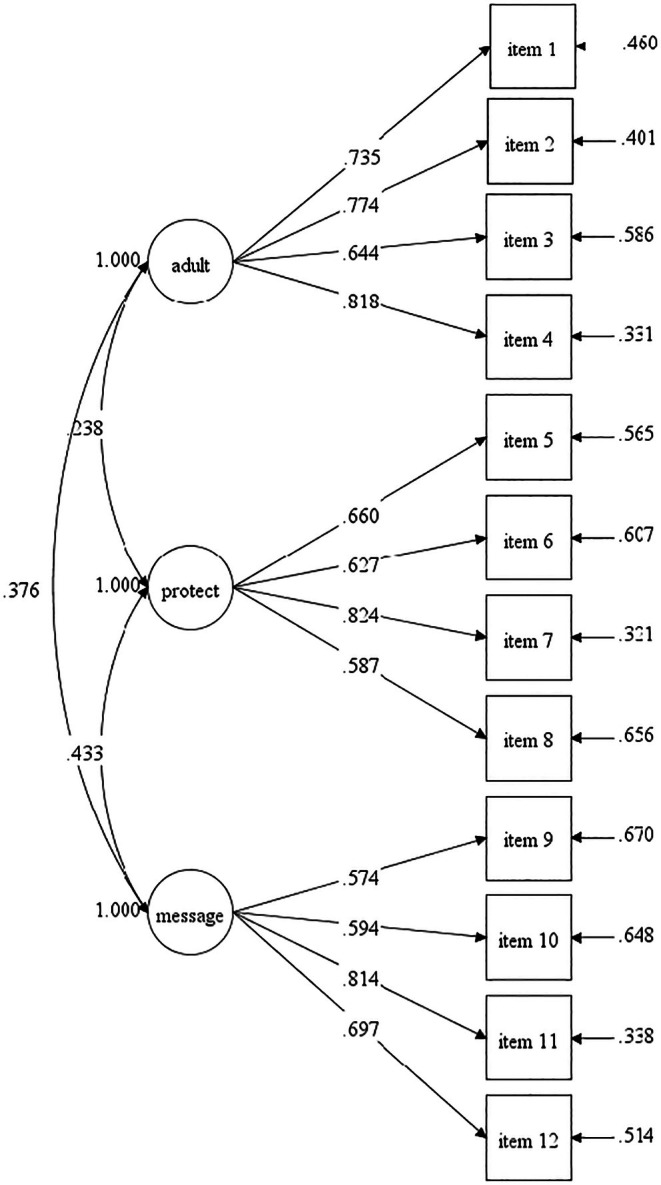
Confirmatory factor analysis highlighting the 12‐item, three‐factor solution. Standardized factor loadings and error terms are presented.

The RAMP subscales were intercorrelated in the expected directions. The adultification subscale was significantly and positively correlated with the protection (*r* = .66), and messaging subscales (*r* = .32) at the *p* < .01 levels. The more participants endorsed being treated like adults, the more they endorsed feeling their bodies were protected, having supportive guidance from parents regarding their bodies, and viewing media as a positive influence on their body image and behavior. RAMPS total was significantly and negatively associated with the adultification scale (*r* = −.43), and significantly and positively associated with the protection and messaging subscales at the *p* < .01 level. Adultification, a measure of sexual risk, was reverse‐scored in the RAMPS total to reflect an overall measure of sexual risk protection; however, it was positively correlated with the protection and messaging subscales, as both require a level of pubertal development and the perception of sexual maturity to be applicable (Figure 2). See Table 2 for the factor intercorrelations.

**TABLE 2:** Factor intercorrelations.

	Adultification	Protection	Messaging
Adultification	–		
Protection	.15*	–	
Messaging	.32*	.35*	–
Total	−.43*	.66*	.56*

* Statistical significance at <.05.

### Measurement invariance

Measurement invariance of RAMPS was examined across age groups (i.e., ages 9–12; 13–15; 16–18; see Table 3). First, the configural invariance model tested whether the pattern of findings was the same across age groups. The configural model fit the data well, and all items loaded significantly onto their corresponding factor. Next, a metric invariance model constraining the factor loadings to be equivalent across groups showed no significant decrement in model fit (*χ* ^2^ = 24.05, *p* > .05). Finally, a scalar invariance model that constrained the factor loadings and item intercepts to be equivalent across groups, while freeing the factor means, also showed no decrement in model fit (*χ* ^2^ = 22.55, *p* > .05). Thus, the interpretation of the measure was fully invariant across age groups.

**TABLE 3:** Measurement invariance across age groups.

Invariance stages	RMSEA	CFI	SRMR
Configural invariance	0.077	0.920	0.058
Metric invariance			
Overall model fit	0.074	0.917	0.073
Change from configural	0.003	0.003	−0.015
Scalar invariance			
Overall model fit	0.071	0.915	0.073
Change from metric	0.003	0.002	0.000

*Note*: Analysis considered three age groups: ages 9–12, 13–15, and 16–18.

Abbreviations: CFI, comparative fit index; RMSEA, root‐mean‐square error of approximation; SRMR, standardized root‐mean‐square residual.

### Construct validity

Associations between RAMPS, including the Adultification, Protect, and Message subscales and related constructs, were assessed. When examining the adultification subscale independently, the scale was left in its original format to indicate higher levels of adultification. However, to acquire a total score for RAMPS, the Adultification subscale is reverse‐scored such that higher scores indicate less adultification. See Table 4 for correlations for all measures.

**TABLE 4:** Correlations between RAMPS and construct validity measures.

	Adultification	Protection	Messaging	Total
BAS‐2 body appreciation	0.08	0.43*	0.12*	0.26*
ACSBI‐S sexual behavior	0.33*	0.03	0.30*	−0.06
Friendship	0.23*	0.18*	0.30*	0.12*
Emotional support	0.06	0.39*	0.10*	0.23*
Maternal	0.03	0.38*	0.04	0.22*
Paternal	0.12*	0.25*	0.12*	0.12*
PDS – puberty	0.15*	0.11*	0.11*	0.02
MIBI‐T centrality	0.14*	0.37*	0.26*	0.26*
MIBI‐T private regard	0.08	0.36*	0.13*	0.22*
MIBI‐T public regard	0.26*	0.26*	0.25*	0.11*

*Note*: For the Adultification subscale, higher scores indicate a greater experience of adultification. For the total score, adultification is reverse‐scored such that a higher score reflects less adultification.

* Correlation is significant at the .05 level (2‐tailed).

RAMPS was positively associated with subscales of the MIBI‐T, including the Centrality (*r* = .26, 95% CI [0.15, 0.37]), Private Regard (*r* = .22, 95% CI [0.09, 0.34]), and Public Regard subscales. This indicates that the more protection participants indicated on RAMPS, the more they viewed race as a central component of their identity, and the more they endorsed positive views of themselves as a member of their race and felt others viewed Blacks positively. Several MIBI‐T subscales were significantly associated with RAMPS subscales in the expected directions (see Table 4).

The RAMPs measure was also positively associated with the BAS‐2 (*r* = .26, 95% CI [0.15, 0.38]), indicating that the more participants endorsed RAMPS, the more positively they viewed their bodies. RAMPS was positively associated with NIH Toolbox measures, including Emotional Support (*r* = .23, 95% CI [0.14, 0.31]) and the Maternal (*r* = .22, 95% CI [0.13, 0.32]) and Paternal Relationship (*r* = .12, 95% CI [0.03, 0.21]) scales. This indicates that the more protection participants indicated on RAMPS, the more they perceived having greater emotional support and closer, more meaningful relationships with their mothers and fathers. The RAMPS measure did not correlate with the ACSBI‐S total score or the PDS as expected.

#### Adultification

Responses on the 4‐item Adultification measure were significantly and positively correlated with MIBI‐T Centrality (*r* = .14, 95% CI [0.06, 0.23]) and Public Regard (*r* = .26, 95% CI [0.17, 0.35]) subscales, indicating that the more participants reported being told or perceiving themselves as appearing or acting older than their age or treated as an adult, the more they viewed race as a central component of their identity, and the more they felt others viewed Black Americans positively. This association is in the expected direction and suggests that a strong racial identity may amplify awareness of societal stereotypes that contribute to feelings of being adultified. Positive correlation with Public Regard suggests that adolescents who believed others held positive views of Black Americans were more likely to report experiences of adultification. This result aligns with our expectations and may reflect the internalization of societal perceptions influencing self‐concept. Adultification was positively correlated with Nationalism (*r* = .11, 95% CI [0.02, 0.19]), which supports that strong pro‐Black attitudes may be related to higher perceptions of adultification, possibly due to increased engagement with cultural norms emphasizing maturity and responsibility. Although not hypothesized, Assimilation (*r* = .28, 95% CI [0.20, 0.36]), Humanism (*r* = .1918, 95% CI [0.09, 0.26]), and Oppressed Minority (*r* = .14, 95% CI [0.50, 0.23]) were also associated with adultification, suggesting that beliefs in universal human similarity or shared oppression may be related to being or feeling adultified. This subscale was not significantly correlated with Private Regard.

Adultification was significantly and positively correlated in the expected direction with the ACSBI‐S total score (*r* = .34, 95% CI [0.28, 0.40]) and each subscale: Sexual knowledge (*r* = .32, 95% CI [0.24, 0.38]), Sexual Risk (*r* = .32, 95% CI [0.26, 0.38]), Divergent Sexual Interests (*r* = .28, 95% CI [0.20, 0.34]), Concern (*r* = .2629, 95% CI [0.21, 0.36]), and Fear (*r* = .26, 95% CI [0.19, 0.33]), indicating that the more participants felt they were treated as grown, the more sexual risk they endorsed. We expected to see that adultification is correlated with risk because Black girls are often adultified and treated as though they are knowledgeable or already engaging in sex. Adultification has been conceptualized as a major contributor pushing Black girls out of schools, which is considered a central driver of sexual exploitation (Morris, 2016; Sissoko et al., 2023). Adultification was positively correlated with PDS, indicating that the more participants felt treated like an adult, the more they endorsed pubertal maturity. The Adultification scale positively correlated with NIH Toolbox Paternal Relationship (*r* = .13, 95% CI [0.03, 0.21]) and Friendship scales (*r* = .23, 95% CI [0.14, 0.32]), indicating that the more participants endorsed being treated like an adult, the closer they felt to their fathers and friends. The Adultification scale did not significantly correlate with the BAS‐2, Maternal Relationships, or Emotional Support.

#### Protection

Responses on the 4‐item Protect subscale were significantly and positively correlated with the MIBI‐T Centrality (*r* = .37, 95% CI [0.28, 0.47]), Private Regard (*r* = .36, 95% CI [0.26, 0.46]), and Public Regard (*r* = .26, 95% CI [0.16, 0.36]) subscales, indicating that the more participants felt their body was protected and had supportive communication and guidance from parents regarding their bodies, the more they viewed race as a central component of their identity, and felt others viewed Blacks positively, and the more they endorsed positive views of themselves as a member of their race. Adolescents who see race as central to their identity and hold positive feelings about being Black report higher levels of familial protection. These findings suggest that a strong racial identity may enhance familial protective factors through reinforced cultural values and open communication within the family. Additionally, positive correlations with Humanism (*r* = .17, CI [0.08, 0.26]) and Nationalism (*r* = .27, 95% CI [0.15, 0.36]) indicate that various dimensions of racial identity may be linked to familial protective behavior. Although not hypothesized, Oppressed minority ideology was also associated with familial protection (*r* = .32, 95% CI [0.22, 0.41]).

Protection was significantly and positively correlated with BAS‐2 (*r* = .43, 95% CI [0.34, 0.53]), indicating that the more participants felt their body was protected, the more positively they viewed their bodies. Protection was significantly and positively associated with the Sexual Knowledge subscale of the ACSBI‐S (*r* = .09, 95% CI [−0.01, 0.18]), indicating that the more protected participants felt their bodies were, the more sexual knowledge they endorsed. Protection was not significantly correlated with the other subscales of the ACSBI‐S or the total measure.

Protection was significantly and positively associated with NIH Toolbox subscales, including Emotional Support (*r* = .39, 95% CI [0.30, 0.47]), the Maternal (*r* = .39, 95% CI [0.29, 0.47]), and Paternal Relationship scales (*r* = .25, 95% CI [0.16, 0.34]), indicating that the more participants felt their body was protected and had supportive communication and guidance from parents, the more emotional support they felt and the better relationships with their mothers and fathers they endorsed. The Protection subscale was significantly and positively associated with PDS (*r* = .11, 95% CI [0.04, 0.19]), indicating that the more participants felt protected, the more likely they were to endorse pubertal development. This subscale did not significantly correlate with the Friendship scale.

#### Messaging

Items on the 4‐item Messaging subscale were significantly and positively correlated with the MIBI‐T Centrality, Private Regard (*r* = .26, 95% CI [0.15, 0.35]), and Public Regard (*r* = .25, 95% CI [0.15, 0.34]) subscales in the expected direction, indicating that the more participants reported a positive impact of media on self‐image and behavior, the more they viewed race as a central component of their identity, felt others viewed being Black positively, and the more they endorsed positive views of themselves as a member of their race. This also suggests that adolescents who prioritize race in their self‐concept perceive social and traditional media as having a more positive influence on their behavior and body image. This hypothesized expectation may reflect their ability to critically engage with media that affirms their racial identity. Positive correlations with Private Regard (*r* = .13, 95% CI [0.02, 0.23]), Public Regard (*r* = .25, 95% CI [0.14, 0.33]), Nationalism (*r* = .24, 95% CI [0.14, 0.33]), and Oppressed Minority (*r* = .21, 95% CI [0.12, 0.32]) further support the notion that a strong and positive racial identity influences how media messaging is interpreted. Contrary to our hypothesis, assimilation (*r* = .26, 95% CI [0.16, 0.34]) and humanism (*r* = .17, 95% CI [0.08, 0.25]) were also positively associated with messaging.

Messaging was significantly and positively associated with BAS‐2 (*r* = .12, 95% CI [0.01, 0.22]), indicating that the more participants reported a positive impact of media, the more positively they viewed their bodies. Messaging was significantly and positively associated with the ACSBI‐S total score (*r* = .30, 95% CI [0.22, 0.36]) and the Sexual Knowledge (*r* = .28, 95% CI [0.20, 0.36]), Deviant (*r* = .24, 95% CI [0.17, 0.31]), Sexual Risk (*r* = .25, 95% CI [0.18, 0.32]), Concern (*r* = .26, 95% CI [0.18, 0.34]), and Fear (*r* = .26, 95% CI [0.19, 0.32]) subscales, indicating the more participants reported positive influences from media, the more sexual risk factors they endorsed on the ACSBI‐S scale.

Messaging was significantly and positively associated with NIH Toolbox subscales, including Emotional Support (*r* = .10, 95% CI [0.00, 0.19]) and the Paternal Relationship scale (*r* = .12, 95% CI [0.04, 0.21]), indicating that the more participants felt their body was protected and had supportive communication and guidance from parents, the more emotional support they felt and the better relationships with their fathers they endorsed.

Overall, the significant positive correlations between RAMPS subscales and key dimensions of racial identity support the construct validity of the RAMPS measure. The associations suggest that a strong racial identity may enhance familial protection, influence perceptions of adultification, and shape interpretations of media messaging, all of which are crucial components in understanding and addressing the risks faced by Black girls.

## DISCUSSION

This study described the development of the Risk, Adultification, Messaging, and Protection Scale (RAMPS), a new 12‐item instrument grounded in the *Becoming a Sexual Black Woman* framework. RAMPS was developed to assess complex sociocultural factors, such as adultification, stereotype messaging, and protection, that shape Black girls' sexual development and overall risk profiles (Crooks et al., 2019). Our analyses demonstrate acceptable to very good internal consistency across subscales (α = .76–.81), and the measure shows a robust factor structure across different age groups (9–18 years).

To our knowledge, the RAMPS is the first measure that captures the experiences of adultification among Black girls. The adultification construct assesses the premature assignment of adult roles and expectations to Black girls. Adultification can be conceptualized as a consequence of stereotypes and the lack of protection for Black girls (Crooks et al., 2023; Epstein et al., 2017). Our findings indicate that higher levels of adultification are associated with heightened racial identity markers, which is consistent with studies suggesting that a strong racial identity may increase sensitivity to societal stereotypes (Perry et al., 2016). The experience of being perceived and treated as more mature or “adult‐like” due to discrimination and racial bias forces an increased awareness of Black girls' racial identity and the societal injustices (Crooks, Sosina, et al., 2022). Additionally, as social media use increases, this can provide spaces for cultural affirmation and identity exploration, but it also increases exposure to stereotypes and negative/biased messaging against Black people. Furthermore, our findings of positive associations between adultification and sexual risk indicators underscore how early perceptions of maturity can influence both behavior and self‐concept in ways that elevate risk.

The messaging subscale evaluates the influence of both traditional and social media messages on Black girls' body image and behavior. Our data showed a nuanced relationship: while positive media messages are associated with higher body appreciation, they also correlate with increased sexual risk factors on the ACSBI‐S. This duality is consistent with literature calling for a more in‐depth exploration of media's multifaceted role in adolescent development (Crooks et al., 2023; Rogers, 2021). The BSBW framework particularly highlights how historical, cultural, and media messaging significantly influences Black girls' sexual development by shaping their identity, attitudes, and behaviors; however, they can reinforce messages of empowerment or harmful stereotypes of Black girls (Crooks, King, & Tluczek, 2022). In this context, further research should dissect which aspects of media messaging most significantly affect positive outcomes and potential risks.

The protection subscale reflects the degree to which one feels they have familial support in protecting their body. Positive correlations with measures of emotional support and parental closeness support the validity of this construct. These findings align with prior studies indicating that familial communication and culturally relevant protective practices can mitigate adverse outcomes (Brown, 2008; Buzi et al., 2009; Coakley et al., 2017). The BSBW framework suggests that protection is the most crucial sociocultural factor influencing Black girls' vulnerability to sexual risk and violence; however, who, how, and what those types of protective strategies are matter, as they are not always effective (Crooks et al., 2020, 2024). As such, measuring perceived protection and family communication is critical for understanding risk and promoting healthy sexual development among Black girls. Future research is needed to explore the mediation and moderation effects of protection regarding familial support and specific parental protective strategies, which may potentially buffer against the negative impacts of adultification and media messaging.

In addition to evaluating each subscale individually, the RAMPS could be used as an integrated measure of sexual risk protection with further testing and refinement. This comprehensive score reflects the cumulative impact of these risk and protective factors on Black girls' sexual development. Importantly, the overall measure provides a brief risk protection profile and practical utility for clinical settings. For example, a low total score may indicate an overall sexual risk protection score that can be leveraged in interventions, while discrepancies among subscales may highlight areas for targeted support. Higher adultification and messaging were associated with greater sexual risk behaviors, while protection was linked to stronger parental relationships, emotional support, body appreciation, and positive racial identity. These relationships suggest that adultification may be seen as a clinical marker of vulnerability, whereas protection and positive racial identity reflect relational and cultural strengths that can be reinforced in prevention and intervention contexts. This integrated approach represents a significant advancement in understanding and addressing the complex interplay of risk and protection in Black girls' sexual development. More research is needed to validate the relationship among these three factors to a total sexual risk protection score. These findings have theoretical implications as they tested constructs in the *Becoming a Sexual Black Woman* framework. This study validates and supports qualitative findings that adultification, which is inherent in Black girls' development, protection, and messaging, has a profound influence on their development.

This study has several limitations, highlighting future research opportunities. Although RAMPS did significantly correlate as expected with many related constructs, it did not correlate as expected with all constructs evaluated. Further evaluation is needed to understand the nomological network of adultification, protection, and messaging. Future research should explore additional sociocultural variables, such as colorism and influences of social class, level of education, and incorporate longitudinal or experimental designs to assess predictive validity for outcomes such as HIV/STI risk. We are currently testing this measure in a randomized control trial of 300 Black girls to further evaluate a total risk/protection score (Crooks et al., 2025). Lastly, although our sample spans a broad age range of Black girlhood, further validation in more geographically and socioeconomically diverse populations is warranted.

## CONCLUSION

The current study supports the initial validity and reliability of RAMPS as a measure of sexual development and correlates of adultification, messaging, and protection among Black girls. By integrating multiple sociocultural constructs into a single, comprehensive instrument, this measure is a valuable tool for both research and clinical practice. Future work will further refine the scale and explore its capacity to predict adverse sexual health outcomes, which will ultimately guide more effective intervention strategies for Black girls.

## AUTHOR CONTRIBUTIONS

All listed authors should have contributed substantially to the manuscript and agreed to the final submitted version. Natasha Crooks and Nyssa Snow‐Hill made a substantial contribution to the concept or design of the article; Abigail Bushnell conducted the data analysis; Gina Sissoko contributed to the interpretation of data for the article, and Kimberly Sanker‐Panchal edited the article.

## FUNDING INFORMATION

This work was supported by the University of Illinois Chicago Building Interdisciplinary Research Careers in Women's Health (BIRCWH) funded by the National Institutes of Health Office for Research on Women's Health (Grant no. K12HD101373‐01).

## CONFLICT OF INTEREST STATEMENT

The authors have no relevant financial or nonfinancial interests to disclose.

## ETHICS STATEMENT

The study was approved by the University of Illinois Chicago, on June 20, 2022. Protocol # 2022‐0550.

## PARTICIPANT CONSENT STATEMENT

Individuals under the age of 18 were assented to the study, and parent/guardian consent was obtained.

## Data Availability

The data that support the findings of this study are available from the corresponding author upon reasonable request.
